# Efficacy and safety of cisplatin, dexamethasone, gemcitabine and pegaspargase (DDGP) regimen in newly diagnosed, advanced-stage extranodal natural killer/T-cell lymphoma: interim analysis of a phase 4 study NCT01501149

**DOI:** 10.18632/oncotarget.10124

**Published:** 2016-06-17

**Authors:** Lei Zhang, Sisi Jia, Yangyang Ma, Ling Li, Xin Li, Xinhua Wang, Xiaorui Fu, Wang Ma, Yanru Qin, Wencai Li, Jingjing Wu, Zhenchang Sun, Xudong Zhang, Feifei Nan, Yu Chang, Zhaoming Li, Dandan Zhang, Guannan Wang, Jiaqin Yan, Liping Su, Jinghua Wang, Hongwei Xue, Ken H. Young, Mingzhi Zhang

**Affiliations:** ^1^ Department of Oncology, The First Affiliated Hospital of Zhengzhou University, Zhengzhou, Henan, 450000, China; ^2^ Department of Pathology, The First Affiliated Hospital of Zhengzhou University, Lymphoma Diagnosis and Treatment Center of Henan Province, Zhengzhou, Henan, China; ^3^ Lymphoma Diagnosis and Treatment Center of Henan Province, Zhengzhou, Henan, 450000, China; ^4^ Department of Hematology, Shanxi Province Cancer Hospital, Taiyuan, Shanxi, China; ^5^ Department of Oncology, Nanjing General Hospital of Nanjing Military Command, Nanjing, Jiangsu, China; ^6^ Department of Oncology, The Affiliated Hospital of Qingdao University, Qingdao, Shandong, China; ^7^ Department of Hematology, The University of Texas MD Anderson Cancer Center, Houston, TX, USA

**Keywords:** extranodal natural killer/T-cell lymphoma, DDGP, chemotherapy, efficacy, safety

## Abstract

To explore a more effective treatment for newly diagnosed, advanced-stage extranodal natural killer/T-cell lymphoma, nasal type (ENKTL), we conducted a phase 4 study of the cisplatin, dexamethasone, gemcitabine, pegaspargase (DDGP) regimen. The primary end point was the 2-year progression-free survival (PFS) after the protocol treatment. Secondary endpoints included response rate (RR), overall survival (OS) and median survival time (MST). The interim analysis included data only from March 2011 to September 2013, who received six cycles of DDGP chemotherapy. A total of 25 eligible patients were enrolled. Seventeen patients (17/24, 70.83%) achieved complete response (CR) and four (4/24, 16.67%) achieved partial response (PR), three (3/24, 12.50%) had progressive disease (PD). The RR after treatment was 87.50%. After a median follow-up duration of 24.67 months (range 4-48 months). The 2-year PFS and OS rate were 61.80% (95% CI, 42.00% to 81.60%) and 68.50 % (95% CI, 48.70% to 88.30%), respectively. The MST was 36.55 months (95% CI, 29.41 months to 43.70 months). Grade 3/4 leukopenia occurred in fourteen patients (58.33%) and grade 3/4 thrombocytopenia occurred in eleven patients (45.83%). Twelve patients (50.00%) experienced Activated Partial Phromboplastin Ptime (APTT) elongation and fourteen patients (58.33%) experienced hypofibrinogenemia. In conclusion, DDGP regimen is an effective and tolerated treatment for newly diagnosed, advanced-stage ENKTL. This trial was registered at www.ClinicalTrials.gov as #NCT01501149.

## INTRODUCTION

Extranodal natural killer /T-cell lymphoma, nasal type (ENKTL) is a rare and highly aggressive lymphoid malignancy with distinct clinical characteristics and dismal outcomes. It shows a peculiar geographic predilection for Asian and South American populations [1]. Most patients with ENKTL have localized disease in the upper aerodigestive tract especially nasal cavity, paranasal sinus, and occasionally in the lung, skin, gastrointestinal tract and testis [2–3]. Concomitant chemoradiotherapy is recommended for localized ENKTL [4–5]. There is still a third of ENKTL patients present with advanced disease [6], for those patients, CHOP (cyclophosphamide, doxorubicin, vincristine, and prednisone) and CHOP-like regiments does not improve survival much (median survival of 2−8 months) [7]. L-asparaginase-based regimens such as L-asparaginase, ifosfamide, methotrexate, etoposide, and dexamethasone (SMILE) or L-asparaginase, methotrexate, and dexamethasone (AspaMetDex) or gemcitabine, oxaliplatin, and L-asparaginase (GELOX) chemotherapy had been tried and the curative effect improved than before [8–11]. However, grade 4 neutropenia was observed in 92% of the patients who was treated with SMILE regimen [8], and about 30% patients because the hypersensitivity reactions could not apply L-Asp-based regimens [12, 13]. The best first-line chemotherapy regimen is not yet established at present, clinical trials are recommended in NCCN Guidelines from 2011 to 2015 year [14].

In order to explore a more effective and safety chemotherapy proposal for ENKTL, from 2009, a series of research on the treatment of ENKTL had been made by Lymphoma Diagnosis and Treatment Center of Henan Province, China. We tested the IC50 of a variety of commonly used chemotherapeutics on SNK-6 cells (a kind of NK/T cell lymphoma line) in vitro experiments including vincristine, anthracycline, bleomycin, dacarbazine, platinum, methotrexate, gemcitabine, L-asparaginase, pegaspargase (a pegylated form of L-asparaginase, not only reduce the risk of hypersensitivity reactions but also increase in plasma half-life when compared with L-asparaginase, has been proved safe and effective in patients with acute lymphoblastic leukemia [15]) et al. We found that gemcitabine (0.002ug/ml) has the lowest IC50 value of all the chemotherapeutics. For pegasparaginase and L-asparaginase, the IC50 values were 4.56 IU/ml and 3.28 IU/ml, respectively. The IC50 value of cisplatin (3.47ug/ml) significantly lower than oxaliplatin (11.610ug/ml) and carboplatin (29.03ug/ml). On the basis of extensive analyses, we formulated a novel pegaspargase-based regimen: cisplatin, dexamethasone, gemcitabine, pegaspargase (DDGP) to treat newly diagnosed ENKTL. From August 2010 to May 2012, our center enrolled 12 newly-diagnosed stage II-IV ENKTL patients treated with DDGP regimen, and 100% RR was achieved (Ten patients (10/12, 83.3%) achieved CR and two (2/12, 16.7%) achieved PR) [16]. In addition, Zhiyuan Zhou, Mingzhi Zhang et al. [17] investigated the efficacy and safety of 17 relapsed/refractory ENKTL patients treated with DDGP regimen between July 2011 and December 2012. The RR and the CR rate were 88.2 % and 52.9 %, respectively. The 1-year OS rate and 1-year PFS rate were 82.4% and 64.7 %, respectively. In 2011, we registered the clinical trials on the U.S. National Institutes of Health (The Clinical Trial registration number: NCT01501149; Official Title: A Randomized Controlled Multi-center Clinical Trial on Treatment of Stage III/IV NK/T Cell Lymphoma with DDGP Regiment). At present, the clinical trial is still recruiting. Here, we will report the interim analysis results about the DDGP regimen in the treatment of newly diagnosed advanced-stage ENKTL.

## RESULTS

### Patients

From March 2011 to September 2013, a total of 25 Chinese patients were randomly assigned to DDGP group. Histologic diagnosis of all patients was confirmed as ENKTL by the pathologists of our lymphoma center. The baseline characteristics of DDGP group patients are listed in Table 1.

**Table 1:** Patients characteristics (N=25)

Characteristic	No. of Patients	%
Age, years		
Median	40	
Range	17 to 64	
Sex		
Male	14	56.00
Female	11	44.00
Subtype of ENKL		
UAT-NTCL	24	96.00
NUAT-NTCL	1	4.00
Stage at enrollment		
III	17	68.00
IV	8	32.00
“B” symptoms present	12	48.00
Elevated serum LDH	9	36.00
Performance status		
0	5	20.00
1	14	56.00
2	6	24.00
NK/T-cell PI score		
0-1	0	0.00
2	12	48.00
3	8	32.00
4	5	20.00

The median age was 40 years (range 17 to 64 years), and the male: female ratio was 14:11. At diagnosis 24 patients (96.00%) had upper aerodigestive tract NK/T-cell lymphoma (UAT-NTCL) and one (4.00%) had extra-upper aerodigestive tract NK/T-cell lymphoma (NUAT-NTCL). 17 patients (68.00%) had newly diagnosed stage III disease, and 8 patients (32.00%) had stage IV disease. 12 patients (48.00%) had B symptoms at presentation, and elevated lactate dehydrogenase (LDH) was observed in 9 patients (36.00 %). None of the patients had CNS involvement or bone marrow invasion. Conventional unfavorable factors for the cohort included high prognostic index of 3 to 4 (52.00%).

### Treatment response and survival outcomes

A total of 25 eligible patients were enrolled, a patient lost to follow-up and out of the group after 2 cycles. Twenty patients (80.00%) completed six cycles of DDGP chemotherapy. Three patients received five cycles of DDGP regimen. One patient accepted four cycles. The total cycles of DDGP regimen received by all patients were 141, with a median of 5.64 cycles (range, 2-6 cycles). The efficacy was estimated in all the 24 patients (96.00%) who received the scheduled treatment (in Table 2). One patient died of brain metastases after four cycles. One patient died from septic shock after five cycles of DDGP regimen. One patient died from multiple organ failure (MOF) after five cycles who achieved CR. One patient rapidly progressed after received five cycles. Thirteen (13/24, 54.2%) patients made dose levels reduction by 20% due to IV degree of bone marrow suppression.

**Table 2:** Treatment and the long-term outcome of 25 newly diagnosed, advanced-stage ENKTL patients

No.	Sex	Age	Diagnosis stage	Cycles	Response	PFS(m)	OS(m)	Current status
1	M	56	UAT,III	2	PR	Exclude	Exclude	Exclude
2	M	34	UAT,III	6	CR	48	48	NOD
3	F	62	NUAT,IV	6	CR	18	24	DOD
4	F	62	UAT,IV	6	PR	47	47	NOD
5	F	34	UAT,III	6	CR	47	47	NOD
6	F	26	UAT,III	6	CR	30	46	Live
7	F	47	UAT,IV	6	CR	9	9	DOD
8	M	54	UAT,III	6	CR	41	41	NOD
9	F	26	UAT,III	5	PD	5	11	DOD
10	M	54	UAT,IV	6	CR	13	14	DOD
11	F	29	UAT,III	6	CR	15	37	Live
12	M	54	UAT,III	5	CR	4	4	DUD
13	M	17	UAT,III	6	PR	33	33	NOD
14	F	43	UAT,III	6	CR	32	32	NOD
15	M	44	UAT,III	5	CR	7	7	DUD
16	M	31	UAT,III	6	CR	29	29	NOD
17	M	42	UAT,III	6	PD	6	26	Live
18	M	23	UAT,IV	4	PD	4	6	DOD
19	F	37	UAT,III	6	PR	24	24	NOD
20	M	24	UAT,IV	6	CR	11	11	NOD
21	F	35	UAT,III	6	PR	21	21	NOD
22	M	48	UAT,IV	6	CR	20	20	NOD
23	F	40	UAT,III	6	CR	19	19	NOD
24	M	64	UAT,III	6	CR	18	18	NOD
25	M	18	UAT,IV	6	CR	18	18	NOD

**Abbreviations:** M male, F female, ENKTL extranodal NK/Tcell lymphoma,

UAT upper aerodigestive tract, DDGP gemcitabine, pegaspargase, cisplatin, and dexamethasone, CR complete response, PR partial response, PD progressive disease, PFS progression-free survival, OS overall survival, m month, DOD dead of disease, DUD dead unrelated to disease, NOD no evidence of disease

At the end of treatment, seventeen patients (17/24, 70.83%) achieved CR and four (4/24, 16.67%) got PR, three (3/24, 12.50%) had PD. The RR and CR rate after treatment were 87.50% and 70.83%, respectively.

**Figure 1:**
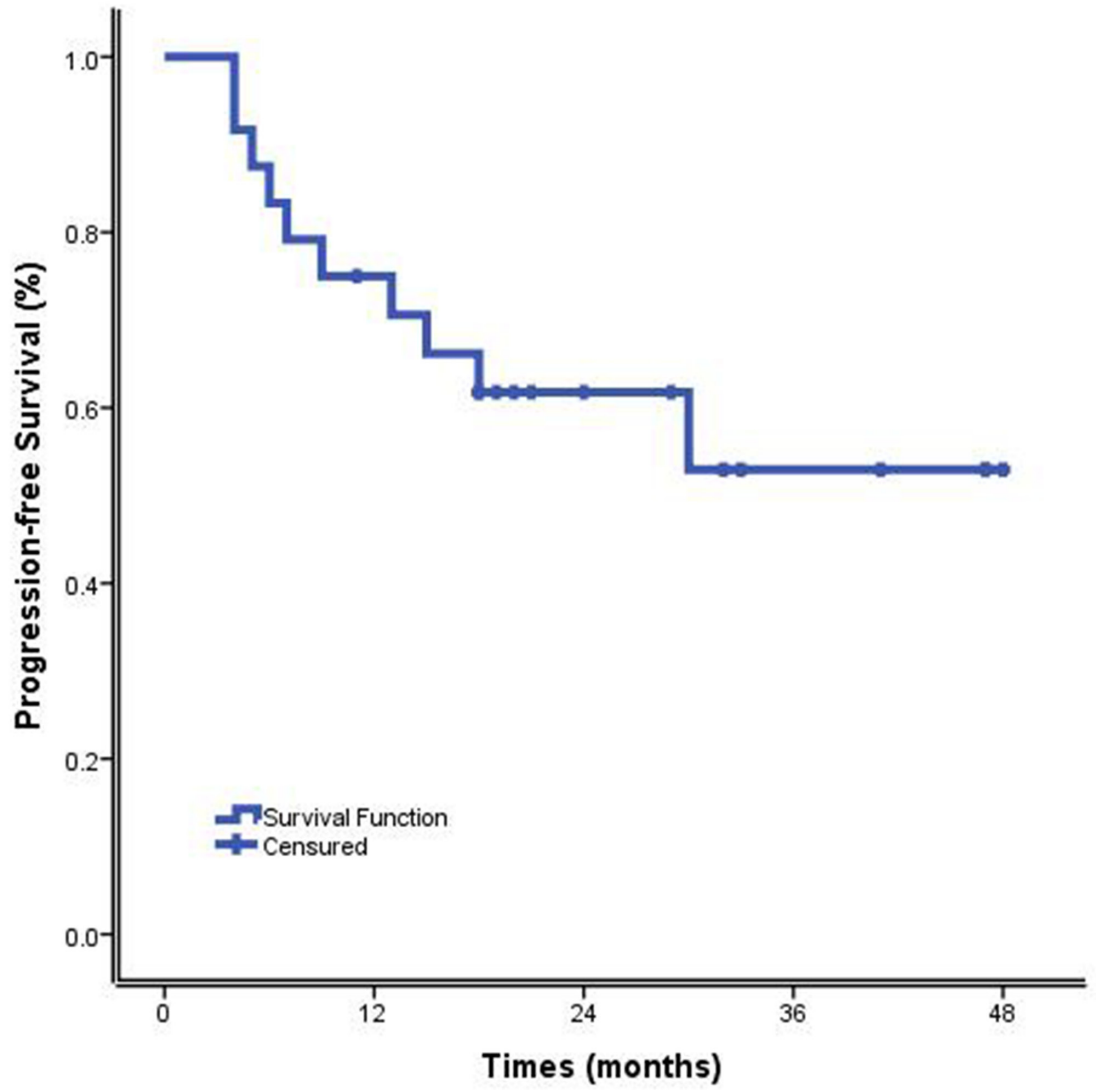
Progression-free survival of cisplatin, dexamethasone, gemcitabine, pegaspargase(DDGP) chemotherapy for patients with newly diagnosed, advanced-stage ENKTL

**Figure 2:**
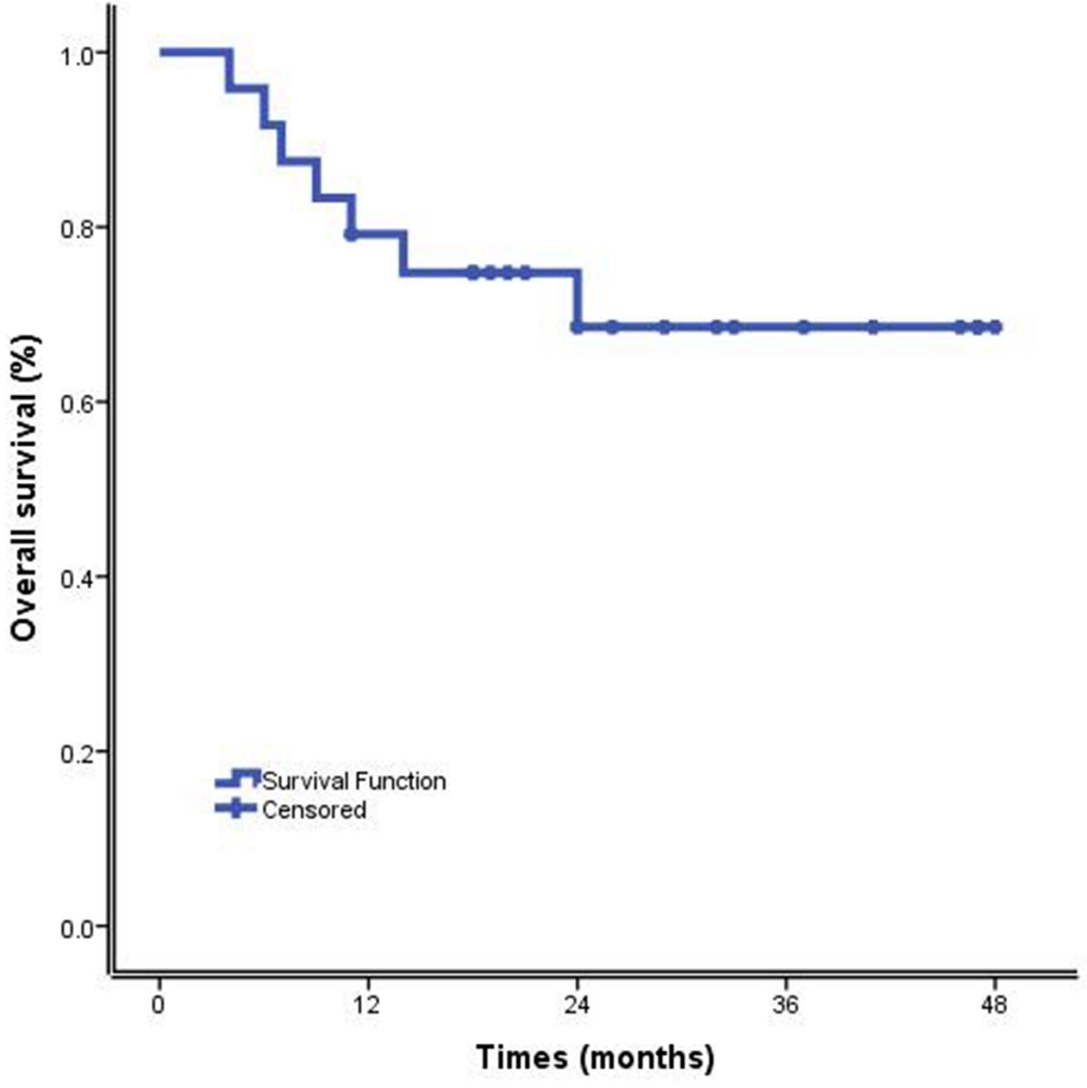
Overall survival of cisplatin, dexamethasone, gemcitabine, pegaspargase (DDGP) chemotherapy for patients with newly diagnosed, advanced-stage ENKTL

By March 2015, fourteen patients (14/24, 58.33%) were alive with no evidence of disease (NOD) after a median follow-up of 24.67 months (range 4-48 months). Five patients (5/24, 20.83%) relapsed at the time of 9, 13, 15, 18 and 30 months after commencing treatment with DDGP regimen, respectively, and two of them were still alive. One of the three patients with disease progression is still alive. Two patients (2/24, 8.33%) died of MOF and septic shock, respectively. The MST was 36.55 months (95% CI, 29.41 months to 43.70 months). The 1-year OS rate and 1-year PFS rate were 79.20% and 75.00%, respectively. The 2-year OS and PFS rate were 68.50 % and 61.8%, respectively. (Figures 1 and 2).

### Assessment of safety

Table 3 lists all grade 1 to 4 adverse events (AEs) that occurred in the 24 eligible patients. The major adverse reactions to DDGP regimen were myelosuppression, digestive tract toxicities, liver dysfunction, and coagulation dysfunction (APTT elongation or decrease in fibrinogen). Grade 3/4 leukopenia occurred in fourteen patients (58.33%) and grade 3/4 thrombocytopenia occurred in eleven patients (45.83%). Twenty-one patients (87.50%) were observed digestive tract toxicities. Twelve patients (50.00%) had APTT elongation and fourteen patients (58.33%) experienced hypofibrinogenemia. There was one patient died from infection which was treatment-related. One patient died from MOF. (See Table 4). No pancreatitis and allergic reaction were seen.

**Table 3:** Treatment-related toxicities

Adverse events	Grade 1/2		Grade3/4	
	No.	%	No.	%
Hematologic				
Leukopenia	9	37.50	14	58.33
Neutropenia	5	20.83	18	75.00
Anemia	14	58.33	9	37.50
Thrombocytopenia	6	25.00	11	45.83
Nonhematologic				
Nausea/vomiting	14	58.33	7	29.17
ALT/AST elevation	14	58.33	1	4.17
Increased BUN	2	8.33	0	--
Heighten of hemodiastase	0			
Increased urinary amylase	0			
Coagulation disorders				
APTT elongation	12 (50.00%)			
Hypofibrinogenemia	14 (58.33%)			

**Table 4:** Seven death patients

No.	Sex	Age	B symptoms	LDH	ECOG	Cycles	Response	Relapse	Treatment after PD/relapse	Death causes
3	F	62	NO	High	2	6	CR	YES	Chemotherapy	lymphoma
7	F	47	NO	Normal	1	6	CR	YES	NO	lymphoma
9	F	26	NO	Normal	0	5	PD	---	RT	lymphoma
10	M	54	YES	High	1	6	CR	YES	RT	lymphoma
12	M	54	NO	Normal	0	5	CR	---	---	Shock*
15	M	44	YES	Normal	2	5	CR	---	---	MOF**
18	M	23	NO	Normal	1	4	PD	---	Alleviative treatment	lymphoma

**Abbreviations**: ECOG Eastern Cooperative Oncology Group, MOF multiple organ failure, RT Radiotherapy

* After five cycles of chemotherapy, this patient experienced grade 4 neutropenia, combination of intestinal infection, led to the deaths of septic shock.

** After chemotherapy, the lymphoma of this patients gradually disappear, but his performance status decreased gradually, finally he died of multiple organ failure.

## DISCUSSION

In our study, we have investigated the use of a pegaspargase-based chemotherapy for the treatment of newly-diagnosed, stage III and stage IV ENKTL, and the results indicate that the efficacy of DDGP regimen was excellent.

The enzyme L-Asp exerts its antitumor effects through the depletion of the essential amino acid L-asparagine, leading to inhibition of protein synthesis in tumor cells [18]. Nagafuji et al. [19] first reported the application of L-Asp in a patient with relapsed nasal NK/T-cell lymphoma after autologous peripheral blood stem cell transplantation. Durable remission was achieved and this patient was alive with NOD after 18 months of follow-up. However, the higher proportion of allergic reaction limiting the use of L-asparaginase [13]. Pegasparaginase, a modified form of L-asparaginase, had greatly reduced the incidence of allergic reaction. Moreover, it has a longer elimination half-life which allows for a considerable reduction in the frequency of drug administration for patients. Reyes Jr V E et al. [20] reported that two patients with relapsed/refractory ENKTL received single-agent pegaspargase, and both entered CR.

Gemcitabine is a kind of cell cycle specificity anti-tumor drugs, mainly killing the tumor cells in S phase (DNA synthesis), as well as blocking the transition process of cell proliferation from G1 phase to S phase. Gemcitabine proved to be effective in pretreated patients with peripheral T-cell lymphoma, especially cutaneous T-cell lymphoma, even in the long term, which can provide rationale for moving it to the frontline therapy [21, 22]. However, because of the rarity of ENKTL, the efficacy of gemcitabine in the treatment of ENKTL is not well known. A retrospective study of gemcitabine-containing regimen for refractory or relapsed ENKTL indicated that gemcitabine was effective in a subset of pretreated ENKTL patients and can be considered as a salvage option [23]. Our previous work confirmed that gemcitabine (0.002ug/ml) has the lowest IC50 value of all the chemotherapeutics, and 100% ORR was observed in our reliminary experiments [16].

In this study, our interim analysis showed a CR rate of 70.83%% and a PR rate of 16.67%, giving an ORR of 87.50%. The CR rate was superior than those treated with SMILE regimen [8, 24], which was similar to those newly-diagnosed localized disease treated with GELOX followed by radiotherapy (74.1%) [10]. The CR rate and survival were much better than historical data using anthracycline-based chemotherapy [1, 11, 25]. (Table 5)

**Table 5:** Study comparison with recent prospective studies of ENKTL

Author, year	Disease status	No. Of patients	Treat	CR rate	Survival	
					OS	PFS
Mingzhi zhang et al. 2015 (this study)	Newly- diagnosed	Stage III/IV: 25	DDGP	70.83%	79.20% (1-yr) 68.50% (2-yr)	75.00% (1-yr) 61.80% (2-yr)
Lin et al. 2013	Newly- diagnosed	Stage I/II: 30 Stage III/IV: 6	CHOP-L ± RT	90% 50%	88.3% (2-yr) 50% (2-yr)	89.5% (2-yr) 50% (2-yr)
Wang L, et al. 2013	Newly -diagnosed	Stage IE/IIE: 27	GELOX± RT	74.1%	86% (2-yr)	86% (2-yr)
Kwong YL, et al. 2012	Newly-diagnosed Relapse/refractory	Newly diagnosed Stage I/II:17 Stage IV: 26 relapse/refractory: 44	SMILE	66%	Newly diagnosed 47.4% (5-yr)	Newly diagnosed 60.0% (4-yr)
Yamaguchi M, et al. 2011	Newly-diagnosed Relapse/refractory	Newly-diagnosed Stage IV: 20; relapse/refractory: 18	SMILE	45%	55% (1-yr)	---
Jaccard et al. 2011	Relapse/refractory	19	AspaMetDex	61%	---	Median PFS: 12.2 months

With regard to the safety of DDGP, the major side effects include bone marrow suppression and coagulant function abnormality. About half of the patients experienced grade 3 and 4 neutropenia. More than half of the patients have high grade thrombocytopenia. To avoid severe adverse events, the use of Granulocyte colony-stimulation factor (G-CSF), recombinant human thrombopoietin (TPO) or Interleukin-11 (IL-11) is essential. Other support treatments such as infection prevention and platelet transfusion were also necessary when patients were in leukopenia and thrombocytopenia status during or after chemotherapy. In case of grade 4 adverse events, doses of chemotherapy agents were reduced to 80% of their previous levels in the next course. In our study, about half of patients experienced 1/2 level of liver dysfunction (ALT/AST elevation) and coagulation dysfunction (prolonged APTT and hypofibrinogenemia). Fortunately, no patients died of severe liver damage or coagulation dysfunction, which may be caused by pegaspargase. However, liver function and coagulation function still need to be closely monitored. In case of hypofibrinogenemia caused by pegaspargase, fresh frozen plasma (FFP), cryoprecipitate or human fibrinogen should be timely applied. Also, glutathlone or compound glycyrrhizin injection could be used to support liver protection therapy. The usual frequency of allergic reaction to pegaspargase is about 2%. However, no allergic reaction to pegaspargase occurred in our study.

In conclusion, this prospective study demonstrates that DDGP is an effective and tolerated chemotherapy for patients with newly diagnosed, advanced-stage ENKTL and indicates the potential of this regimen as a first-line therapy against this disease. But the small sample size, and short follow-up period suggest that our findings need to be further investigated with a larger patient group and longer follow-up period. At present, our clinical trial is still recruiting to evaluate its efficiency and safety.

## MATERIALS AND METHODS

### Study design

This was an open label, randomized, controlled, multi-center, phase 4 study of DDGP chemotherapy treated with newly diagnosed advanced-stage ENKTL. The study was approved by the Human Subjects Committee and institutional review board at each participating institution, and all patients signed an informed consent document describing the investigational nature of the proposed treatment. The primary end point was the 2-year PFS after the protocol treatment. Secondary outcome measures included RR, OS and MST. The PFS was measured from the date of treatment until disease progression or death from any cause or the last follow-up. The RR was defined as the proportion of all patients who experienced CR and PR. OS was defined as the time from treatment until death from any cause or the last follow-up. MST refered to the survival time when the cumulative survival rate was 50 percent. The study was conducted in accordance with the Declaration of Helsinki and was registered with www.ClinicalTrials.gov as #NCT01501149.

Eligible patients were randomly assigned by the study coordinating center to treatment with DDGP or Modified SMILE alone. Patients treated with DDGP received the combination of 20 mg of cisplatin per square meter of body-surface area on days 1-4, 15 mg of dexamethasone per square meter on days 1–5, 800 mg of gemcitabine per square meter on day 1 and day 8, and 2,500 IU of pegaspargase per square meter on day 1. They were treated every 21 days for six cycles of DDGP. Patients treated with Modified SMILE received methotrexate 2 g/m^2^ (6 hours), dexamethasone 40 mg/d d2-4, ifosfamide 1,500 mg/m^2^ d2-4, L-asparaginase 6,000 U/m^2^ d3-9, etoposide 100 mg/m^2^ d2-4, Mesna 300 mg/m^2^ d2-4. Cycles were repeated every 21 days. Six courses were planned as the protocol treatment.

The doses of chemotherapy drugs were reduced to 80% of their previous dose levels in patients who had grade 4 adverse events, except for pegaspargase. Tumor responses were assessed every two cycles of chemotherapy. During the treatment, patients were routinely monitored for blood tests, serum chemistry, and coagulation function. G-CSF and TPO or IL-11 were given to patients who developed neutropenia and thrombocytopenia as support therapy. The patients were withdrawn from the study when they meet any conditions as follows: patients' conditions deteriorated so as to transfer to other treatments immediately including disease progression; patients experienced severe and intolerant toxicity reaction; chemotherapy delayed more than two weeks; patients asked for dropping out of the study or the researchers thought the medical need to withdraw from this study.

### Patients

Inclusion Criteria were as follows: (1) Age range 14-70 years old; (2) ECOG performance status 0-2; (3) Estimated survival time > 3 months; (4) Histological confirmed NK/T cell lymphoma, nasal-type according to WHO classification [26]; (5) The NSS [27] stage III or IV; (6) None of chemotherapy or radiotherapy has been previously used; (7) None of chemotherapy contraindication: hemoglobin ≥ 90 g/dl, neutrophil ≥ 1.5×10^9^/L, platelet ≥ 100×10^9^/L, ALT and AST ≤ 2×upper limitation of normal (ULN), serum bilirubin ≤ 1.5×ULN, serum creatine ≤ 1.5×ULN, Serum Albumin ≥ 30g/L, serum plasminogen is normal; (8) At least one measurable lesion; (9) None of other serious diseases, cardiopulmonary function is normal; (10) Pregnancy test of women at reproductive age must be negative; (11) Patients could be followed up; (12) None of other relative treatments including the traditional Chinese medicine, immunotherapy, biotherapy except anti-bone metastasis therapy and other symptomatic treatments; (13) Volunteers who signed informed consent.

The exclusion criteria included: (1) Disagreement on blood sample collection; (2) Patients allergic of any of drug in this regimen or with metabolic disorder; (3) Pregnant or lactating women; (4) Serious medical illness likely to interfere with participation; (5) Serious infection; (6) Primitive or secondary tumors of central nervous system; (7) Chemotherapy or radiotherapy contraindication; (8) The evidence of CNS metastasis; (9) History of peripheral nervous disorder or dysphrenia; (10) Patients participating in other clinical trials; (11) Patients taking other antitumor drugs; (12) Patients estimated to be unsuitable by investigator.

After patients enrollment, their pathological data were reviewed by pathologists based on the WHO classification [16]. Proven NK/T-cell type by immuneohistochemistry (cytoplasmic CD3ε+, CD20-phenotype, a cytotoxic profile, and markers of EBV by in situ hybridization).

Clinical stage based on the NSS which was established by Tongyu Lin [27]. Stage III was defined localized disease with regional lymph node involvement (cervical lymph nodes); and stage IV: disseminated disease (lymph nodes on both sides of diaphragm, multiple extranodal site). B symptoms were defined as unexplained fever with temperature above 38°C, night sweats, and unexplained weight loss of more than 10% of the usual body weight in the 6 months before diagnosis. The NK/T-cell prognostic index score includes B symptoms, Ann-Arbor stage III/IV, elevated serum LDH level, and regional lymphadenopathy [28].

### Assessment of efficacy and adverse events

The responses were assessed according to criteria modified from the 2010 NCCN response criteria about non-Hodgkin's lymphoma [29]. CR was defined as no evidence of disease. PR was defined as a reduction of at least 50% of the pretreatment tumor burden without the occurrence of new lesions at the restaging. PD was defined as a greater than 50% increase in the sum of tumor lesions or the emergence of one or more new lesions or clinical symptoms that indicate disease progression. Stable disease (SD) was defined as any response that did not fall into the other defined categories.

All adverse events were assessed at each cycle from the first day of the regimen until one month after the last treatment and graded according to the National Cancer Institute Common Toxicity Criteria, Version 3.0.

### Statistical analysis

The SPSS Statistics 21.0 software was used to data analysis. The Kaplan-Meier method was used to perform survival analysis for OS, PFS and MST. The 95% CI were calculated for PFS and OS. All CIs reported were 2-sided.

Toxicity was monitored continuously throughout the study, with descriptive statistics provided of observed events. Toxicities reported were the worst-grade toxicities per patient during the induction period and during the maintenance period.
